# Differentiating impacts of non‐pharmaceutical interventions on non‐coronavirus disease‐2019 respiratory viral infections: Hospital‐based retrospective observational study in Taiwan

**DOI:** 10.1111/irv.12858

**Published:** 2021-04-07

**Authors:** Andrew Po‐Liang Chen, Isaac Yen‐Hao Chu, Mei‐Lin Yeh, Yin‐Yin Chen, Chia‐Lin Lee, Hsiao‐Hsuan Lin, Yu‐Jiun Chan, Hsin‐Pai Chen

**Affiliations:** ^1^ Division of Infectious Diseases Department of Medicine Taipei Veterans General Hospital Taipei Taiwan; ^2^ Institute of Epidemiology and Preventive Medicine National Taiwan University Taipei Taiwan; ^3^ Department of Public Health, Environments and Society Faculty of Public Health and Policy School of Hygiene and Tropical Medicine London United Kingdom; ^4^ Nursing Department Taipei Veterans General Hospital Taipei Taiwan; ^5^ Department of Infection Control Taipei Veterans General Hospital Taipei Taiwan; ^6^ Institute of Public Health School of Medicine National Yang‐Ming University Taipei Taiwan; ^7^ Division Microbiology Department of Pathology and Laboratory Medicine Taipei Veterans General Hospital Taipei Taiwan; ^8^ School of Medicine National Yang‐Ming University Taipei Taiwan

**Keywords:** COVID‐19 pandemic, enterovirus, enveloped respiratory viruses, infection control, Influenza virus, non‐enveloped respiratory viruses, non‐pharmaceutical intervention, seasonal coronavirus

## Abstract

**Background:**

Physical distancing and facemask use are worldwide recognized as effective non‐pharmaceutical interventions (NPIs) against the coronavirus disease‐2019 (COVID‐19). Since January 2020, Taiwan has introduced both NPIs but their effectiveness on non‐COVID‐19 respiratory viruses (NCRVs) remain underexplored.

**Methods:**

This retrospective observational study examined electronic records at a tertiary hospital in northern Taiwan from pre‐COVID (January–December 2019) to post‐COVID period (January–May 2020). Patients with respiratory syndromes were tested for both enveloped (eg, influenza virus and seasonal coronavirus) and non‐enveloped RVs (eg, enterovirus and rhinovirus) using multiplex reverse transcription polymerase chain reaction assays. Monthly positivity rates of NCRVs among adult and pediatric patients were analyzed with comparison between pre‐ and post‐COVID periods.

**Results:**

A total of 9693 patients underwent 12 127 multiplex RT‐PCR tests. The average positivity rate of NCRVs reduced by 11.2% (25.6% to 14.4%) after nationwide PHIs. Despite the COVID‐19 pandemic, the most commonly identified enveloped and non‐enveloped viruses were influenza virus and enterovirus/rhinovirus, respectively. Observed reduction in NCRV incidence was predominantly contributed by enveloped NCRVs including influenza viruses. We did not observe epidemiological impacts of NPIs on non‐enveloped viruses but an increasing trend in enterovirus/rhinovirus test positivity rate among pediatric patients. Our data were validated using Taiwan's national notification database.

**Conclusions:**

Our frontline investigation suggests that the current NPIs in Taiwan might not effectively control the transmission of non‐enveloped respiratory viruses, despite their protective effects against influenza and seasonal coronavirus. Health authorities may consider using hydrogen peroxide or chloride‐based disinfectants as additional preventative strategies against non‐enveloped respiratory viruses in the post‐COVID‐19 era.

## 1 INTRODUCTION

While the coronavirus disease‐2019 (COVID‐19) continues casting global health burdens, non‐COVID‐19 viral respiratory tract infections (RTIs) continue devastating millions of lives with estimated 4 million deaths worldwide.^1^, ^2^ Prior to the severe acute respiratory syndrome coronavirus‐2 (SARS‐CoV‐2), the most commonly diagnosed pathogenic respiratory viruses are influenza virus, parainfluenza virus (PIV), seasonal coronavirus (sCoV), enterovirus (EnV) and rhinovirus (RhV), adenovirus (AdV), human metapneumovirus (hMPV), and human bocavirus (hBoV). These viruses can also be virologically classified into two groups: enveloped viruses (eg, influenza virus, PIV, and sCoV) and non‐enveloped viruses (eg, AdV, EnV, and RhV). Without implementing a combination of timely testing, accurate diagnosis, effective treatment, and non‐pharmaceutical interventions, countries’ healthcare systems could be heavily exhausted by these viral RTIs compounded with the COVID‐19 pandemic.^3^, ^4^, ^5^, ^6^

Physical distancing and face mask use have been worldwide recognized as effective non‐pharmaceutical interventions (NPIs) to mitigate the spread of COVID‐19. Mathematical models have forecast that an 80% coverage of face mask use among populations can effectively reduce the transmission and mortality of SARS‐CoV‐2 by 17%‐45%.^7^ A Chinese study using real‐world data reported that face mask use in COVID‐19 patients and their close contacts resulted in a 79% risk reduction of SARS‐CoV‐2 transmission.^8^ A global systematic review reported that face mask use and physical distancing could reduce risks of SARS‐CoV‐2 infections by 85% and 82%, respectively.^9^ Using N95 respirators among medical practitioners could reduce the infection rate of COVID‐19 by 95%.^10^ Together with contact tracing systems, such measures have been implemented by countries worldwide to combat the COVID‐19 pandemic.

Taiwan, one of the countries with the lowest incidence rate and mortality of COVID‐19, has become an exemplar in effectively implementing NPIs. Following the first COVID‐19 case reported in Wuhan, China on December 31, 2019, Taiwan confirms its first COVID‐19 case on January 22, 2020 in Taiwan. Taiwan Center of Disease Control (TCDC) has implemented different levels of NPIs since the inception of COVID‐19 pandemic. Individuals must wear facemasks in public transportations, healthcare facilities, and indoor public space. Physical distancing was also strictly requested at restaurants and populated public venues. Moreover, Taiwanese governments strictly executed international border control by requesting all arriving passengers a 14‐day compulsory quarantine with active surveillance on respiratory symptoms and body temperature. After adopting these NPIs, TCDC observed a decline in positivity rates of influenza virus—an enveloped virus as SARS‐CoV‐2—declined from 375 cases in January 2020 to zero after March 2020.^11^ A similar epidemiological change was also reported during the 2003 SARS‐CoV epidemic in Taiwan where the number of diagnosed RTIs plummeted after the introduction of NPIs from January 2003 to April 2003.^11^ Increasing facemask use, awareness of personal hygiene screening seeking behavior was observed during the 2003 SARS‐CoV epidemic in Hong Kong.^12^ Despite the observed decline of influenza infection during SARS and COVID‐19 epidemics, little is known about the epidemiological impact of COVID‐19‐related NPIs on the positivity rates of non‐COVID‐19 RVs (NCRVs) (eg, influenza virus, sCoV, and EnV). Whether test positivity rates of enveloped and non‐enveloped viruses vary by NPIs remains uncertain. To understand the potential impacts of NPIs on the incidence of NCRVs, we examined the change in positivity rates during the COVID‐19 pandemic in Taiwan.

## 2 METHODS

### 2.1 Study design and patient recruitment

We presented a retrospective cross‐sectional study using hospital‐based surveillance data from Taipei Veterans General Hospital (TVGH), one of the biggest medical centers in Taiwan. We examined medical records on patients presenting respiratory symptoms from January 2019 to May 2020, defining two periods as pre‐COVID (January 2019 to December 2019) and post‐COVID (January 2020 to May 2020). The study was approved by the institutional review board of TVGH (reference number: 2019‐06‐022CC).

### 2.2 Respiratory examinations

Patients who presented respiratory symptoms were queried for traveling, occupation, contact, and cluster (TOCC) history followed by physical examinations and chest radiogram. One set of nasopharyngeal swab sample was collected from patients and sent to a centralized Biosafety Level 2 laboratory for multiplex reverse transcription polymerase chain reaction (RT‐PCR)‐based assays. The RT‐PCR assays were run by the Luminex xTAG® Respiratory Virus Panel (Luminex Molecular Diagnostics) or the BIOFIRE® FILMARRY® Respiratory Panel (BioFire Diagnostics). Types of RVs detectable by the two RT‐PCR panels included AdV, influenza virus, PIV, hMPV, sCoV, RSV, and EnV/RhV (Appendix 1).

### 2.3 Levels of non‐pharmaceutical interventions

Levels of NPIs in Taiwan were escalated with an increasing epidemic curve for COVID‐19 from January 2020. Here, we focused on the efficacy of interventions such as the use of personal protective equipment and physical distancing. The Central Epidemic Command Center enforced serial regulations on the use of personal protective equipment. The level 1 facemask regulation (F1) was adopted on January 28, 2020, when the first imported COVID‐19 case was confirmed in Taiwan. The government released two million facemasks into the market while restricting exports of medical facemasks; the general public were requested to wear facemask in crowded public space and at healthcare facilities. Meanwhile, a facemask rationing plan allowed Taiwanese to purchase up to two medical facemasks weekly per person with the registration of National Health Insurance cards. The level 2 facemask regulation (F2) was implemented on February 11, 2020, by which the government provided facemasks to public transportation drivers, and patients and caregivers in medical facilities on a daily basis. The level 3 facemask regulation (F3) was implemented on March 5, 2020, which allowed personal purchases for up to five facemasks per 14 days. The regulation also requested all passengers on public transportation wearing a facemask compulsorily with a fine at up to $2,000 USD on violators.

Taiwan officials announced physical distancing regulation (P) on March 25, 2020. Indoor activities involving over 100 participants, and outdoor activities involving over 500 participants were banned. The ban also discouraged unnecessary travels to reduce potential contacts. Most public activities were postponed or canceled after the announcement of the ban.

### 2.4 Data analysis

Given the average incubation periods and serial intervals of NCRVs span from one to two weeks,^13^, ^14^, ^15^, ^16^, ^17^ those who were tested more than once in any 14‐day period were only counted as one test. Overall positivity rates of NCRVs each calendar month were calculated. The positivity rate of each NCRV was calculated to examine trend changes with various NPIs. We also categorized NCRVs into enveloped and non‐enveloped viruses based on their virologic characteristics. Positivity rates of these two virological groups were examined against different NPIs in a temporal sequence. Due to the limited sample size, we regrouped AdV, hMPV, hBoV, and PIV as other viruses. National surveillance data from TCDC on influenza virus infection were applied to examine data validity and representativeness.

### 2.5 Statistical analysis

Descriptive statistics were applied to describe patient characteristics. Categorical variables were analyzed by Pearson's chi‐squared test. The significance level was set at 0.05. All analyses were conducted using RStudio^®^ statistical software (version: 1.3.959).

## 3 RESULTS

Of all 9693 patients undergoing 12 127 multiplex RT‐PCR tests, 4855 were tested from January to December 2019 and 4838 from January to May 2020. Table 1 lists patient characteristics. The proportion of adult (aged 18 and above) patients were 84.4% (4099/4855) and 94.0% (4546/4838) in pre‐COVID and post‐COVID period, respectively. Compared with the same period in 2019, the total number of examined patients from January to May 2020 increased by 2.9 times (4838 versus 1664), with a 3.4‐fold increase (4546 versus 1334) in adult visits and an 11% decrease (292 versus 330) in pediatric visits. The proportion of patients infected with more than one NCRVs during pre‐COVID period were higher than those visited during post‐COVID period (3.7% versus 1.4%).

**TABLE 1:** Characteristics of patients being tested for non‐COVID respiratory viruses from January 2019 to May 2020

	2019 (Pre‐COVID)	2020 Jan to May (Post‐COVID)
Total number of RT‐PCR tests	6012	6115
Number of repetitive tests (%)	1157 (19.2%)	1277 (20.1%)
Number of patients	4855	4838
Adult (%)	4098 (84.4%)	4546 (94.0%)
Pediatric (%)	757 (15.6%)	292 (6.0%)
Median Age	64	62
Adult patient	69	64
Pediatric patient	4	5
Number of patients infected with at least one NCRV (%)	1242 (25.6%)	696 (14.4%)
Number of patients infected with two or more NCRVs (%)	179 (3.7%)	66 (1.4%)
Number of patients under intensive care (%)	1079 (22.2%)	497 (10.3%)

Abbreviations: NRCV, Non‐COVID‐19 Respiratory Virus; RT‐PCR, reverse transcription polymerase chain reaction.

### 3.1 Data validation

**FIGURE 1:**
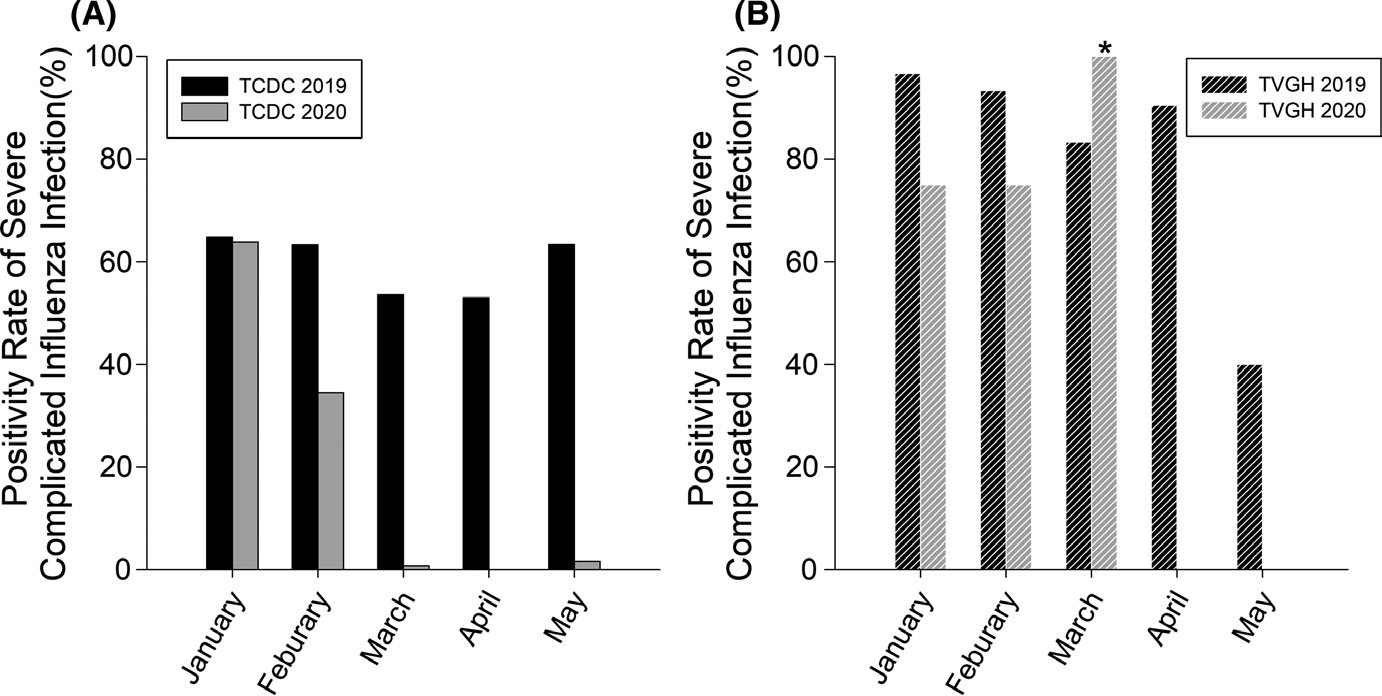
Positivity Rates of severe complicated influenza infection reported either by Taiwan Center of Disease Control (TCDC) and Taipei Veterans General Hospital (TVGH). A, the black sold bars were the rates that TCDC reported in 2019 while the white bars were those in 2020. B, the black stripe bars were the rates that TVGH reported in 2019 while the white stripe bars were those in 2020. Rates of severe influenza virus infection were defined as the number of patients with respiratory failure and confirmed with influenza virus infection by reverse transcription polymerase chain reaction (RT‐PCR) tests divided the total number of patients with respiratory failure and proceeded RT‐PCR tests. *The outlier in March 2020 TVGH reported was 100% due to that there was only one patient suspected with influenza‐related respiratory failure and confirmed by RT‐PCR test

According to Taiwan National Infectious Disease Statistics System (TNIDSS), positivity rates of influenza virus in patients suspected with severe complicated influenza infection (defined as those with respiratory failure) were around 40% nationwide from January 2019 to January 2020, followed by a steady decline from February 2020 when NPIs were implemented to April 2020 when no cases of severe influenza infection were reported (Figure 1A). The trend of positivity rates of influenza in patients with severe respiratory tract infections at TVGH corresponded with TCDC’s national statistics during pre‐COVID and post‐COVID periods, except for March 2020 given a relatively small sample size.^11^ Specifically, only one case suspected with severe complicated influenza infection was notified at TVGH in March 2020 with subsequent RT‐PCR confirmation, resulting in a 100% case positivity rate (Figure 1B).

### 3.2 Overall positivity rates of NCRVs

**FIGURE 2:**
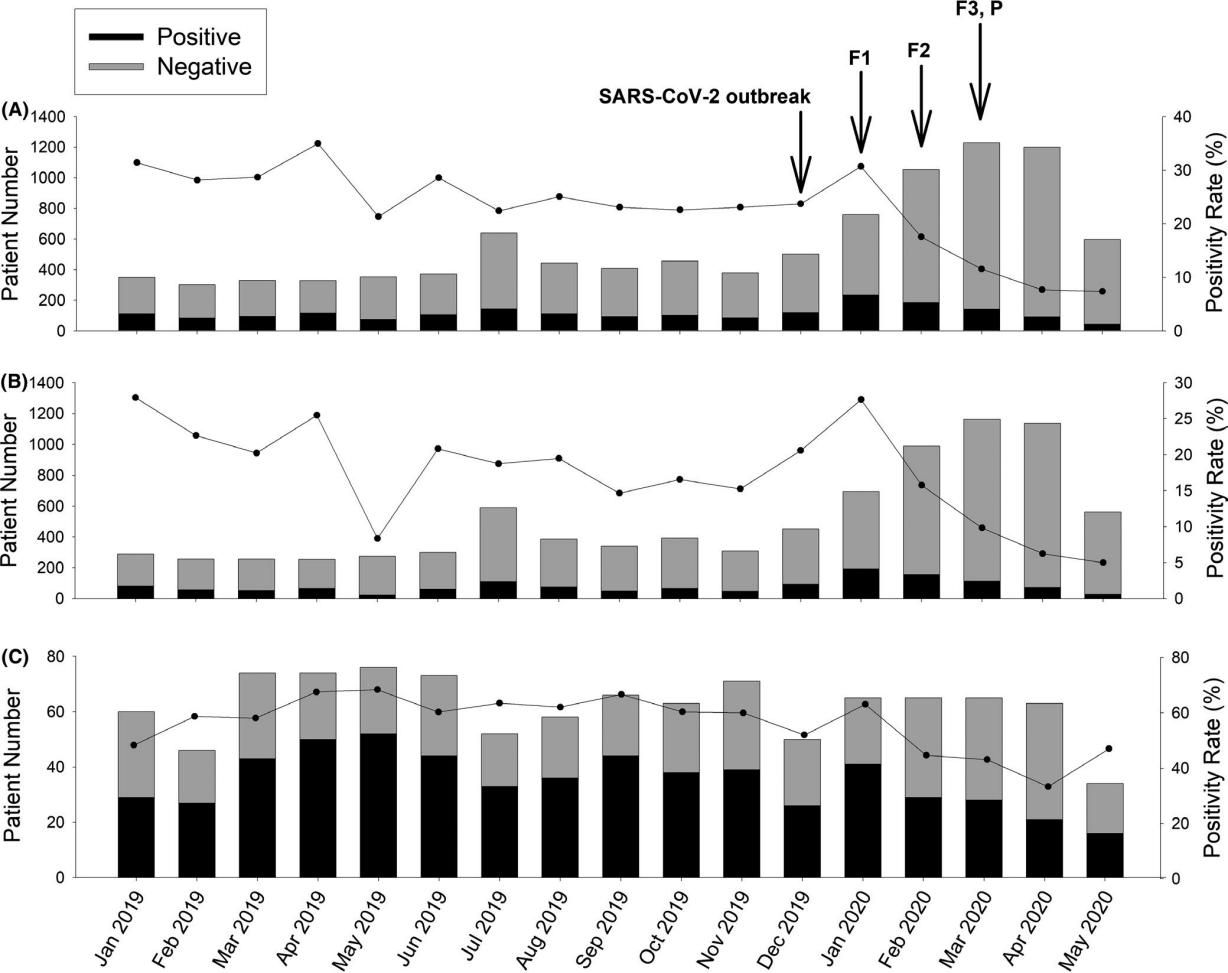
Number of patients and positivity rates of non‐COVID respiratory viruses. A, All patients. B, Adult patients. C, Pediatric patients. SARS‐CoV‐2 broke out in January 2020 and the Taiwanese government implemented various non‐pharmaceutical interventions and infection control measures since February 2020. F1: level 1 facemask regulation, started on January 28. F2: level 2 facemask regulation, started on February 11. F3: level 3 facemask regulation, started on March 3. P: physical distancing, started on March 25

**FIGURE 3:**
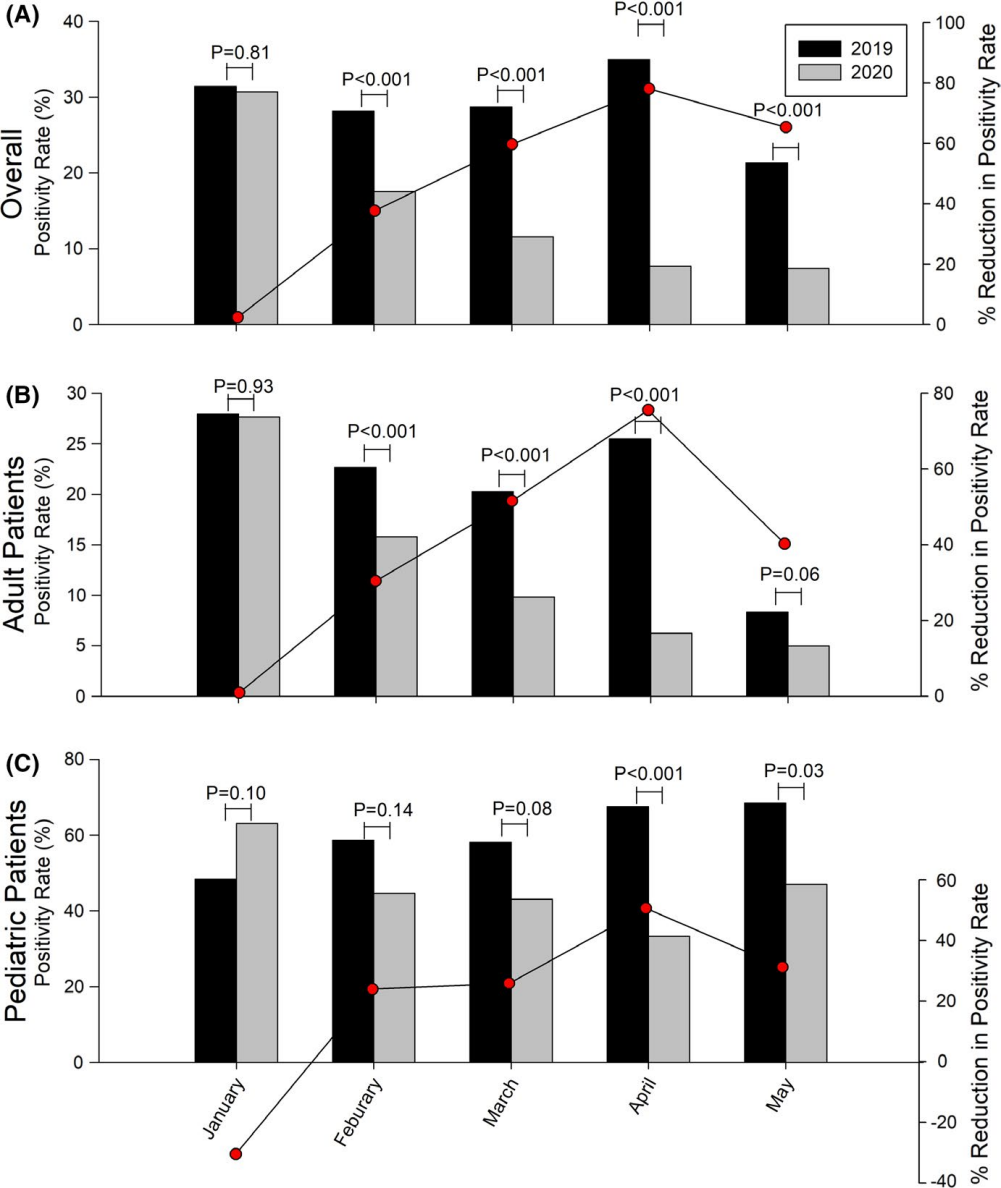
Comparison of monthly positivity rates and rates of reduction of non‐COVID‐19 respiratory viruses between January and May 2019 and January and May 2020, with reduction of positivity rates (the red spots). Percentage reduction of positivity rate was defined as positivity rate in 2020 subtracted that of 2019 and divided by positivity rate in 2019. 3A, all patients. 3B, adult patients. 3C, pediatric patients

The average positivity rate of NCRVs was 25.6% pre‐COVID and 14.4% in post‐COVID period. While the trend of NCRV incidence remained steady in pre‐COVID time, the rate peaked in January 2020 at 30.7% and declined gradually after Taiwan introduced NPIs as from 17.6% in February to 7.4% in May 2020 (Figure 2A). Compared to the same period in 2019, the positivity rate in January 2020 was similar, but those from February to May 2020 were significantly lower (Ps<0.001) with a 17.2% average rate of reduction (Figure 3A). In adult patients, positivity rates from February 2020 to April 2020 reduced by 10.0% than the same months in 2019 (Figure 2B). We also observed a 3.3% rate reduction in May 2020 compared with that of May 2019 (5.0% versus 8.3%, *P* =.06), albeit the presence of stringent NPIs (Figure 3B). Among pediatric patients, the average positivity rate of NCRVs in the study period was 56.3%. The positivity rates in both April 2020 and May 2020 were lower than the same months in 2019 following the implementation of F1‐F3 and P phase of NPIs from Mach 2020 (Figure 2C). The average rate of reduction was 21.2%, reflecting a 48.6% alteration compared with the previous year (Figure 3C). When the Taiwanese government leveled up facemask regulations from F1 to F3, a higher degree of positivity rate reduction was observed (See Appendix 2 for detail).

### 3.3 Positivity rates of enveloped and non‐enveloped respiratory viruses

**FIGURE 4:**
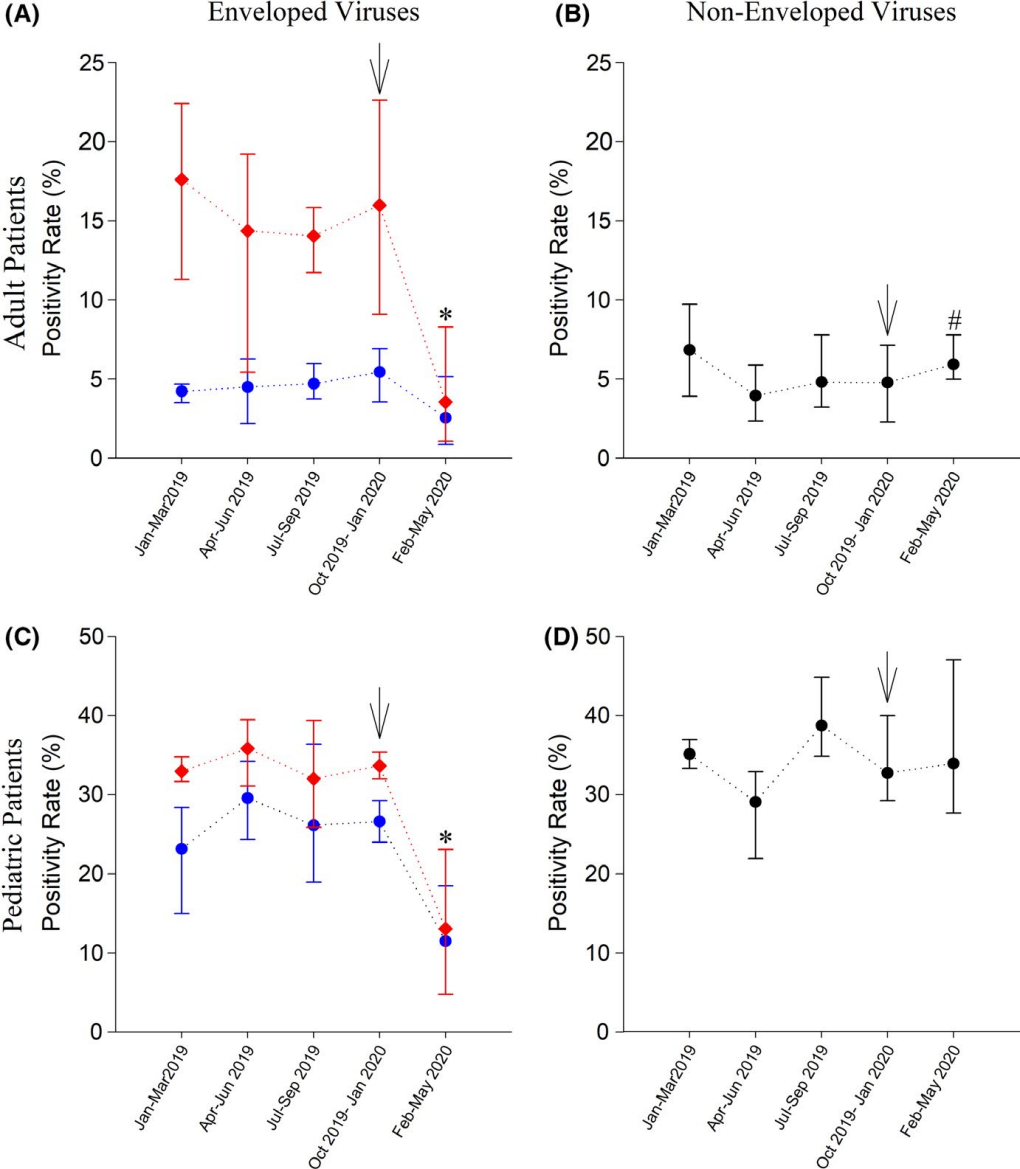
Average positivity rates of enveloped and non‐enveloped non‐COVID‐19 respiratory viruses (NCRVs) in five different periods since 2019. A, Enveloped NCRVs in adult patients. B, Non‐enveloped NCRVs in adult patients. C, Enveloped NCRVs in pediatric patients. D, Non‐enveloped NCRVs in pediatric patients. Red line: data include influenza virus; blue line: positivity rates of enveloped NCRVs, pure influenza virus infection was excluded. Black arrow: sequential non‐pharmaceutical interventions implemented by the Taiwanese government from January 28. *:*P* <.001; #:*P* =.04

The influenza virus was the most predominant enveloped NCRVs in both adult (59.0%±22.7%) and pediatric (20.4%±14.3%) patients. The average positivity rate of enveloped NCRVs in adults in pre‐COVID time was 14.0%‐17.6% (Figure 4A, red line). Such rate remained stable (4.2%‐5.4%) exclusive of influenza virus (Figure 4A, blue line). After a combination of NPIs was implemented in January 2020, the average positivity rate of non‐influenza enveloped NCRVs in adults dropped to 3.6% in February‐May 2020 (Figure 4A). A similar drop was observed in pediatric patients at 2.5% (Figure 4C). Adjusting for influenza virus infection, the positivity rates in both adult (2.5% versus 4.7%, *P* <.001) and pediatric patients (11.5% versus 26.4%, *P* <.001) reduced significantly after NPIs implementation (Figure 4A, C). Considering the potential effects of NPIs on non‐enveloped NCRVs, the average positivity rates did not decline after the implementation of NPIs in Taiwan. In adults, compared with the pre‐COVID period, a 0.8% increase in the average positivity rate was shown after initiation of the NPIs (5.9% versus 5.1%, *P* =.04) (Figure 4B). The positivity rate in children remained still regardless of the implementation of NPIs (33.9% vs 33.8%, *P* =.69) (Figure 4D).

### 3.4 Positivity rates of influenza virus

**FIGURE 5:**
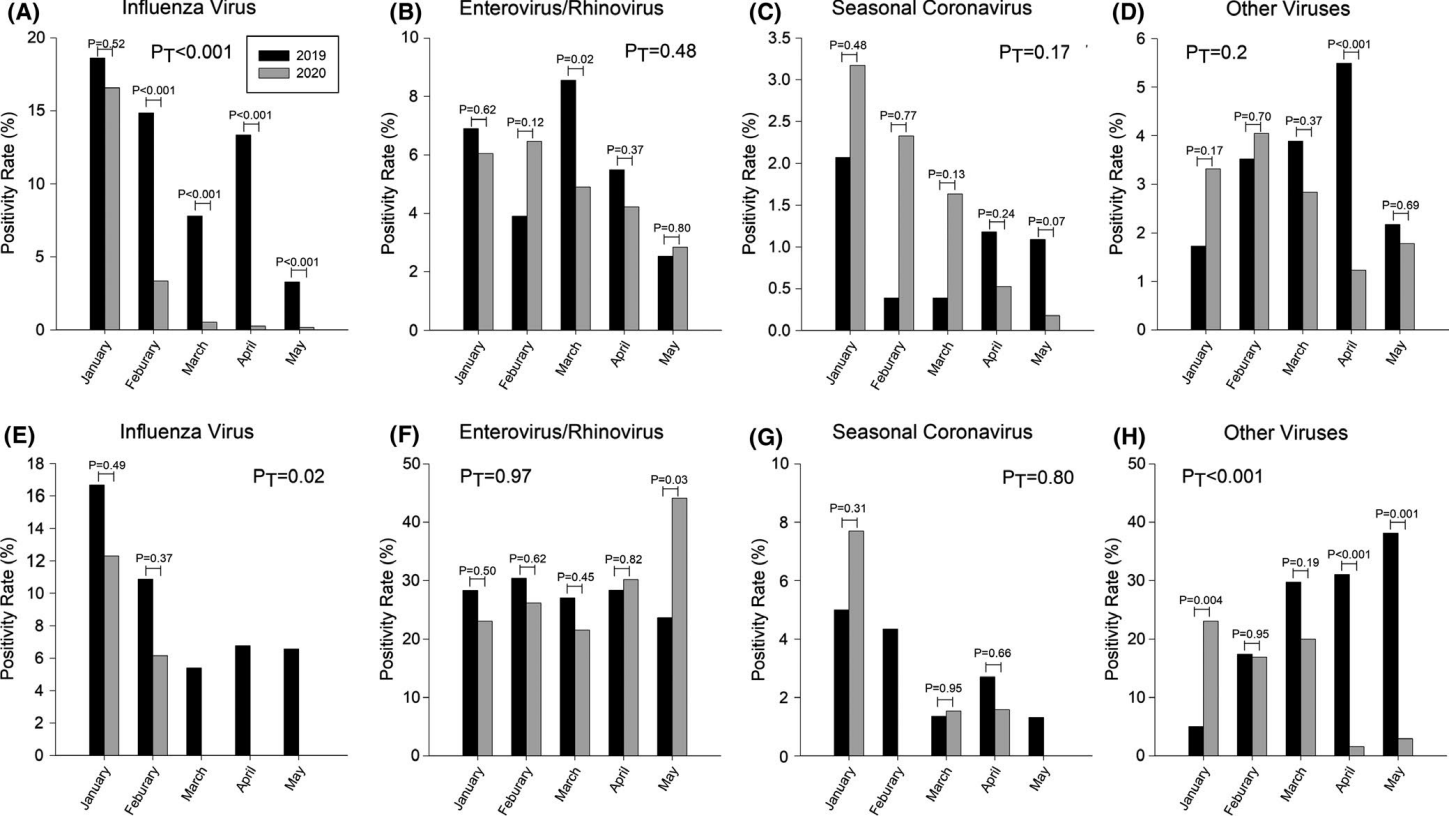
Positivity rates of influenza virus, enterovirus/rhinovirus, seasonal coronavirus, and other viruses. A, B, C, and D, Adult patients; E, F, G, and H, Pediatric patients. Due to limited patients in adenovirus, parainfluenza virus, human metapneumovirus, human bocavirus, and respiratory syncytial virus, we categorized these viruses as “other viruses” in this study. P_T_: p values which compared overall positivity rates of four different non‐COVID respiratory viruses in January 2020 to May 2020 with those of the same period in 2019

During the early phase of COVID‐19 epidemic in January 2020, the positivity rate of influenza in TVGH was comparable to that in 2019. As the government started strengthening the intervention measures, the positivity rate of adult patients declined from 16.6% in January 2020 to 3.3% in February 2020, and the overall positivity rate from January to May in 2020 was significantly lower than that in 2019 (P_T_<0.001, Figure 5A). There was no influenza pediatric case observed after March 2020, and the overall positivity rate in 2020 was significantly lower than that in 2019, which was similar to adult patients (P_T_=0.02, Figure 5E).

### 3.5 Positivity rates of Enterovirus/Rhinovirus (EnV/RhV)

Regardless of NPIs, the positivity rates of EnV/RhV during the COVID‐19 epidemic did not reduce as compared to the previous year. In adults, the positivity rate of EnV/RhV remained above 6% in January and February 2020, and then gradually declined after March 2020, but the monthly positivity rates remained similar to those of the previous year. The overall positivity rate of adult patients in 2020 (4.9%) was not significantly different than that in 2019 (5.5%) (P_T_=0.48, Figure 5B). However, in pediatric patients, even with stepwise NPIs, we did not observe a significant decline in the positivity rates of EnV/RhV during the epidemic period. On the other hand, the positivity rate increased above 30% since April 2020 and became significantly higher in May 2020 (44.1%) as compared to May 2019 (23.7%) (*P* =.03, Figure 5F). The overall positivity rate of pediatric patients in 2020 (29.0%) was also not significantly different than that in 2019 (27.6%) (P_T_=0.97).

### 3.6 Positivity rates of seasonal coronaviruses (sCoVs)

A high level of positivity rates was observed during the early phase of COVID‐19 epidemic since January 2020. In adult patients, the positivity rates of sCoVs in 2020 were higher than that in the same period in 2019, and the overall positivity rate in 2020 (1.6%) was higher than that in 2019 (1.0%). Although the positivity rates of 2020 did not reach the statistically significant difference than those of 2019 in adult patients, the positivity rates during post‐COVID period were still higher, especially from January to March (P_T_=0.17, Figure 5C). A peak of positivity rate was observed in pediatric patients in January 2020; the overall positivity in 2020 (2.2%) was lower than that in 2019 (2.9%) (P_T_=0.80, Figure 5G). The positivity rates gradually declined with the implementation of higher levels of NPIs. Regarding subtypes of sCoVs in adults, sCoV‐229E was predominant during the pre‐COVID period (20/41, 48.8%), followed by sCoV‐OC43 (16/41, 39.0%), and sCoV‐229E (5/41, 12.2%); in the post‐COVID period, sCoV‐OC43 became the most common subtype (35/71, 49.3%), followed by sCoV‐229E (18/71, 25.4%), sCoV‐NL63 (13/71, 18.3%), sCoV‐HKU1 (4/71, 5.6%), and one case with an undetermined subtype. Considering pediatric patients, sCoV‐OC43 was predominant during the pre‐COVID period (14/25, 56.0%), followed by sCoV‐229E (5/25, 20.0%), sCoV‐229E (4/25, 16.0%), and sCoV‐HKU1 (2/25, 8.0%); in the post‐COVID period, sCoV‐OC43 (3/7, 42.9%) and sCoV‐NL63 (3/7, 42.9%) were both commonly detected in children, followed by sCoV‐229E (1/7, 14.2%).

### 3.7 Positivity rates of other respiratory viruses

With respect to all other NCRVs, compared with the same period in 2019, the positivity rates in adult patients rose in January 2020 then gradually declined after March 2020, and the positivity rate in April 2020 (1.2%) was significantly lower than that in April 2019 (5.5%) (*P* <.001). However, the overall positivity rate in 2020 (2.6%) was not significantly different than that in 2019 (3.4%) (P_T_=0.2, Figure 5D). Similarly, the positivity rate in pediatric patients in January 2020 was much higher than that of the previous year (23.1% to 5.0%, *P* <.001), but it declined since April 2020 to a value lower than that in 2019; and, the overall positivity rate in 2020 (12.9%) was significantly lower than of that in 2019 (24.3%) (P_T_<0.001, Figure 5H).

## 4 DISCUSSION

Our study revealed a three‐fold increase in the numbers of patients tested for NCRVs after the COVID‐19 outbreak in January 2020. Regarding types of NCRVs, influenza virus and enterovirus/rhinovirus (EnV/RhV) were the most commonly reported enveloped and non‐enveloped viruses regardless of the COVID‐19 pandemic. While the overall test positivity rate of NCRVs reduced after TCDC introduced NPIs nationwide, such reduction was predominantly contributed by enveloped NCRVs. We did not observe the epidemiological impacts of NPIs on non‐enveloped viruses in our hospital‐based research.

Our findings have consolidated the protective effects of NPIs against enveloped viruses, including influenza viruses and seasonal coronaviruses. The results are in line with other studies of the effectiveness of NPIs (ie, facemask usage and physical distance) on controlling NCRVs in both the 2003 SARS outbreak in Taiwan and the current COVID‐19 pandemic.^13^, ^14^ During the 2003 SARS outbreak, the positivity rates of NCRVs, especially influenza virus, dropped significantly after Taiwanese governments enacted NPIs such as universal facemask wearing and body temperature monitoring.^18^ Recent studies have witnessed a reduction in the incidence of influenza viruses after NPIs were adopted in Taiwan, Japan, New Zealand, and the United States. ^14^, ^15^, ^16^, ^17^ Regarding sub‐population differences, we found that test positivity rates for influenza in adults dropped one month earlier than children (ie, February 2020 versus March 2020). The temporal difference may be attributed to varied facemask availability between adults and children. Specifically, adult‐size facemasks have been widely available since 28 January 2020, but child size ones were not available nationwide until 5 March 2020. More research in how and to what extent, availability of personal‐protection equipment affects the mortality and mobility of sub‐populations caused by both NCRVs and COVID‐19 are warranted.

Our results provide compelling evidence that current NPIs may have limited impacts on combating non‐enveloped NCRVs. Contrary to existing findings from TNIDSS and the National Health Insurance Research Database, our hospital‐based research showed that test positivity rates in both adults and children for EnV/RhV remained stagnant.^19^ The observed differentiated impacts of NPIs on enveloped and non‐enveloped viruses can be explained by three factors. Firstly, non‐enveloped viruses are more resistant to environmental challenges (eg heat, desiccation, and pH values) than enveloped viruses. The former are hydrophilic and with more extended survival periods than the latter with lipid bilayer, treated with alcohol‐based disinfectants.^20^, ^21^ Although Taiwan has adopted alcohol‐based fumigation to combat COVID‐19 transmission, the resistant nature of non‐enveloped viruses make both enteroviruses and rhinoviruses survive longer and thereby increase their likelihoods of transmission via person‐to‐person or contaminated surface.^22^, ^23^, ^24^, ^25^, ^26^, ^27^ Secondly, the protective effect of face mask may depend on types of viruses. Leung et al’s randomized control trial suggests that face mask is more effective in filtering out enveloped viruses (eg, influenza virus and sCoV) than non‐enveloped viruses (eg, rhinovirus).^28^ This proposition can help to explain a rhinovirus outbreak in New Zealand, albeit its stringent interventions against COVID‐19.^29^ Thirdly, while Taiwan government requests all citizens to wear facemasks and improve hand hygiene, most venues only provide alcohol‐based hand sanitizer, which had little viricidal effects against non‐enveloped viruses. One can still carry and spread non‐enveloped viruses after touching contaminated surfaces. Given alcohol‐based disinfectants cannot kill non‐enveloped viruses, chloride‐ and hydrogen peroxide‐based products should be added into guidance for comprehensive infection control.^30^ While keeping increasing the public awareness of hand hygiene against non‐enveloped viruses, governments should apply a combination of disinfectants to infection control so that the negative consequences of both the COVID‐19 and NCRVs pandemics can be further mitigated.

One notable observation from our study was that the positivity rates of sCoVs in January‐March 2020 were much higher than the same periods in 2019. Such increase in sCoVs positivity rate might be explained by co‐incidental COVID‐19 outbreak. Nevertheless, the positivity rates declined as NPIs were gradually intensified. Studies have shown that facemask usage and physical distancing were effective in preventing the transmission of sCoVs..^31^ However, studies demonstrating the correlation between sCoV incidence and individual NPI remained scarce. Further studies are necessary to address such issues in the post‐COVID‐19 era.

Our study bears several limitations. Firstly, our research was conducted in a medical center in northern Taiwan with a limited number of patients. Our results, particularly of pediatric patients, cannot be generalized to national epidemiological trends of viral infection in Taiwan. We urge more studies using multi‐center primary data and follow‐up with more pediatric patients to capture the real‐world epidemiological changes. Secondly, RT‐PCR tests were not commonly used in clinical assessment in the pre‐COVID‐19 era. As the number of patients tested for NCRVs increased much compared with situations in 2019, our reported changes in trends might be overestimated. Thirdly, the reliability of specimen collection measures among different physicians was unknown, albeit guidance on the standard of care. Different ways in specimen collection could bias test positivity rates of NCRVs. Moreover, types of testable viruses are limited to the two multiplex RT‐PCR systems, neither of which can distinguish enterovirus from rhinovirus infection. Subjected to such limitations, our test positivity rates of EnV/RhV can be biased and of limited use. Lastly, physical distancing, the universal wearing of masks, and hand hygiene have all been reported to be effective in reducing NCRVs transmission.^16^, ^17^, ^18^, ^19^ While non‐pharmaceutical interventions might have had synergistic effects to prevent transmission of NCRVs, the size and extent of effectiveness should be carefully examined through a systemic approach. We could not calculate the individual effectiveness of every NPI that the Taiwanese government implanted. Factors that potentially confound or interact with each NPI on the incidence of NCRVs should be investigated carefully in future studies applying mathematical modeling or causative designs.

## 5 CONCLUSION

Non‐pharmaceutical interventions play essential roles in preventing respiratory virus transmission. Comprising facemask wearing, physical distancing and alcohol‐based sanitizers and disinfectants, current public health interventions may not be sufficient to mitigate the spread of non‐enveloped viruses. When such outbreaks threaten healthcare capacity, health authorities may consider hydrogen peroxide or chloride‐based disinfectants as additional preventative strategies in the post‐COVID‐19 era.

## CONFLICTS OF INTEREST

No conflict of interest has been declared by the authors.

## AUTHOR CONTRIBUTION

**Andrew Po‐Liang Chen:** Conceptualization (equal); Formal analysis (lead); Investigation (equal); Methodology (equal); Supervision (equal); Writing‐original draft (lead). **Isaac Yen‐Hao Chu:** Validation (lead); Visualization (lead); Writing‐review & editing (lead). **Mei‐Lin** **Yeh:** Data curation (equal). **Yin‐Yin Chen:** Resources (equal). **Chia‐Lin Lee:** Investigation (equal). **Hsiao‐Hsuan Lin:** Investigation (equal). **Yu‐Jiun Chan:** Conceptualization (equal); Resources (equal); Supervision (equal). **Hsin‐Pai Chen:** Conceptualization (equal); Methodology (equal); Supervision (equal).

### PEER REVIEW

The peer review history for this article is available at https://publons.com/publon/10.1111/irv.12858.

## Supporting information

Appendix S1

Appendix S2

## Data Availability

The data that support the findings of this study are available on request from the corresponding author. The data are not publicly available due to ethical restrictions.
