# Supplementary material for: Differentiating impacts of non‐pharmaceutical interventions on non‐coronavirus disease‐2019 respiratory viral infections: Hospital‐based retrospective observational study in Taiwan
Source: Influenza Other Respir Viruses. 2021 Apr 7;15(4):478–87. doi: 10.1111/irv.12858 (PMC8189242; doi:10.1111/irv.12858)
Supplement: Supplementary file 1 — Appendix S1 [file IRV-15-478-s002.docx]

**Supplementary Material 1** List of microorganisms detected by BIOFIRE® FIlMARRY® Respiratory Panel and Luminex xTAG® Respiratory Virus Panel.

|  | BIOFIRE® FIlMARRY®  Respiratory Panel | Luminex xTAG®  Respiratory Virus Panel |
| --- | --- | --- |
| Viruses | 1. Adenovirus 2. Seasonal coronaviruses: OC43, 229E, NL63, HKU1 3. Human metapneumovirus 4. Human bocavirus 5. Enterovirus/Rhinovirus 6. Influenza 7. Parainfluenza 8. Respiratory syncytial virus | 1. Respiratory syncytial virus 2. Influenza 3. Parainfluenza 4. Seasonal coronaviruses: OC43, 229E, NL63, HKU1 5. Human metapneumovirus 6. Enterovirus/Rhinovirus 7. Adenovirus 8. Human bocavirus |
| Bacteria | 1. Bordetella pertussis 2. Bordetella parapertussis 3. Chlamydophila pneumoniae 4. Mycoplasma pneumoniae |  |
