# Supplementary material for: Differentiating impacts of non‐pharmaceutical interventions on non‐coronavirus disease‐2019 respiratory viral infections: Hospital‐based retrospective observational study in Taiwan
Source: Influenza Other Respir Viruses. 2021 Apr 7;15(4):478–87. doi: 10.1111/irv.12858 (PMC8189242; doi:10.1111/irv.12858)
Supplement: Supplementary file 2 — Appendix S2 [file IRV-15-478-s001.docx]

**Supplementary Material 2** Number of patients and test positivity rate of non-COVID-19 respiratory viruses (NCRVs) per month throughout the study period.

**Table 1 Number and test positivity rate of NCRVs among adult patients from 2019 January to 2020 May.**

| Year/  Month | Number of  examined patients | Number of confirmed cases* | Enveloped virus | | | | | | | Non-enveloped virus | | | |
| --- | --- | --- | --- | --- | --- | --- | --- | --- | --- | --- | --- | --- | --- |
|  |  |  | Overall | Influenza | NIE****** | PIV | hMPV | sCoV | RSV | Overall | AdV | EnV/RhV | hBoV |
| 2019/01 | 290 | 81 (27.9) | 65 (22.4) | 54 (18.6) | 13 (4.5) | 2 (0.7) | 2 (0.7) | 6 (2.1) | 3 (1.0) | 20 (6.9) | 0 (0) | 20 (6.9) | 0 (0) |
| 2019/02 | 256 | 58 (22.7) | 49 (19.1) | 38 (14.8) | 12 (4.7) | 3 (1.2) | 4 (1.6) | 1 (0.4) | 4 (1.6) | 10 (3.9) | 0 (0) | 10 (3.9) | 0 (0) |
| 2019/03 | 257 | 52 (20.2) | 29 (11.3) | 20 (7.8) | 9 (3.5) | 4 (1.6) | 4 (1.6) | 1 (0.4) | 0 (0) | 25 (9.7) | 3 (1.2) | 22 (8.6) | 3 (1.2) |
| 2019/04 | 255 | 65 (25.5) | 49 (19.2) | 34 (13.3) | 16 (6.3) | 2 (0.8) | 10 (3.9) | 3 (1.2) | 1 (0.4) | 15 (5.9) | 3 (1.2) | 14 (5.5) | 1 (0.4) |
| 2019/05 | 276 | 23 (8.3) | 15 (5.4) | 9 (3.3) | 6 (2.2) | 1 (0.4) | 2 (0.7) | 3 (1.1) | 0 (0) | 10 (3.6) | 3 (1.1) | 7 (2.5) | 0 (0) |
| 2019/06 | 298 | 62 (20.8) | 55 (18.5) | 41 (13.8) | 15 (5.0) | 8 (2.7) | 5 (1.7) | 1 (0.3) | 1 (0.3) | 7 (2.3) | 3 (1.0) | 4 (1.3) | 0 (0) |
| 2019/07 | 587 | 110 (18.7) | 93 (15.8) | 77 (13.1) | 22 (3.7) | 7 (1.2) | 11 (1.9) | 6 (1.0) | 2 (0.3) | 20 (3.4) | 3 (0.5) | 12 (2.0) | 7 (1.2) |
| 2019/08 | 385 | 75 (19.5) | 56 (14.5) | 35 (9.1) | 23 (6.0) | 6 (1.6) | 3 (0.8) | 6 (1.6) | 10 (2.6) | 30 (7.8) | 1 (0.3) | 14 (3.6) | 19 (4.9) |
| 2019/09 | 341 | 50 (14.7) | 40 (11.7) | 26 (7.6) | 15 (4.4) | 4 (1.2) | 3 (0.9) | 1 (0.3) | 7 (2.1) | 11 (3.2) | 1 (0.3) | 7 (2.1) | 3 (0.9) |
| 2019/10 | 393 | 65 (16.5) | 58 (14.8) | 37 (9.4) | 24 (6.1) | 4 (1.0) | 6 (1.5) | 3 (0.8) | 13 (3.3) | 9 (2.3) | 3 (0.8) | 5 (1.3) | 1 (0.3) |
| 2019/11 | 308 | 47 (15.3) | 28 (9.1) | 14 (4.5) | 16 (5.2) | 7 (2.3) | 5 (1.6) | 5 (1.6) | 0 (0) | 22 (7.1) | 4 (1.3) | 19 (6.2) | 0 (0) |
| 2019/12 | 452 | 93 (20.6) | 79 (17.5) | 64 (14.2) | 16 (3.5) | 5 (1.1) | 4 (0.9) | 5 (1.1) | 2 (0.4) | 15 (3.3) | 5 (1.1) | 10 (2.2) | 0 (0) |
| 2020/01 | 694 | 192 (27.7) | 157 (22.6) | 115 (16.6) | 48 (6.9) | 7 (1.0) | 11 (1.6) | 22 (3.2) | 9 (1.3) | 44 (6.3) | 3 (0.4) | 42 (6.1) | 1 (0.1) |
| 2020/02 | 989 | 156 (15.8) | 82 (8.3) | 33 (3.3) | 51 (5.2) | 19 (1.9) | 8 (0.8) | 23 (2.3) | 4 (0.4) | 77 (7.8) | 12 (1.2) | 64 (6.5) | 3 (0.3) |
| 2020/03 | 1163 | 114 (9.8) | 42 (3.6) | 6 (0.5) | 37 (3.2) | 13 (1.1) | 3 (0.3) | 19 (1.6) | 4 (0.3) | 69 (5.9) | 12 (1.0) | 57 (4.9) | 0 (0) |
| 2020/04 | 1138 | 71 (6.2) | 14 (1.2) | 3 (0.3) | 11 (1.0) | 3 (0.3) | 1 (0.1) | 6 (0.5) | 1 (0.1) | 57 (5.0) | 9 (0.8) | 48 (4.2) | 0 (0) |
| 2020/05 | 562 | 28 (5.0) | 6 (1.1) | 1 (0.2) | 5 (0.9) | 3 (0.5) | 1 (0.2) | 1 (0.2) | 0 (0) | 28 (5.0) | 5 (0.9) | 16 (2.8) | 0 (0) |

(): positivity rate, defined as percentage of confirmed cases of all examined patients. NIE: non-influenza enveloped virus; PIV: parainfluenza; hMPV: human metapneumovirus; sCoV: seasonal coronavirus; RSV: respiratory syncytial virus. AdV: adenovirus; EnV/RhV: enterovirus/rhinovirus; hBoV: human bocavirus.
*: calculated as patients being infected with at least one type of NCRVs.
**: calculated as patients being infected with at least one of the four viruses (PIV, hPMV, sCoV, and RSV).

**Table 2 Number and test positivity rate of NCRVs among pediatric patients from 2019 January to 2020 May.**

| Year/  Month | Number of  examined patients | Number of confirmed cases* | Enveloped virus | | | | | | | Non-enveloped virus | | | |
| --- | --- | --- | --- | --- | --- | --- | --- | --- | --- | --- | --- | --- | --- |
|  |  |  | Overall | Influenza | NIE****** | PIV | hMPV | sCoV | RSV | Overall | AdV | EnV/RhV | hBoV |
| 2019/01 | 60 | 29 (48.3) | 19 (31.7) | 10 (16.7) | 9 (15.0) | 1 (1.7) | 1 (1.7) | 3 (5.0) | 4 (6.7) | 20 (33.3) | 5 (8.3) | 17 (28.3) | 1 (1.7) |
| 2019/02 | 46 | 27 (58.7) | 16 (34.8) | 5 (10.9) | 12 (26.1) | 2 (4.3) | 3 (6.5) | 2 (4.3) | 5 (10.9) | 17 (37) | 5 (10.9) | 14 (30.4) | 0 (0) |
| 2019/03 | 74 | 43 (58.1) | 24 (32.4) | 4 (5.4) | 21 (28.4) | 12 (16.2) | 7 (9.5) | 1 (1.4) | 2 (2.7) | 26 (35.1) | 10 (13.5) | 20 (27) | 1 (1.4) |
| 2019/04 | 74 | 50 (67.6) | 23 (31.1) | 5 (6.8) | 18 (24.3) | 5 (6.8) | 9 (12.2) | 2 (2.7) | 3 (4.1) | 24 (32.4) | 3 (4.1) | 21 (28.4) | 1 (1.4) |
| 2019/05 | 76 | 52 (68.4) | 30 (39.5) | 5 (6.6) | 26 (34.2) | 8 (10.5) | 11 (14.5) | 1 (1.3) | 6 (7.9) | 25 (32.9) | 11 (14.5) | 18 (23.7) | 0 (0) |
| 2019/06 | 73 | 44 (60.3) | 27 (37.0) | 6 (8.2) | 22 (30.1) | 10 (13.7) | 3 (4.1) | 0 (0) | 9 (12.3) | 16 (21.9) | 4 (5.5) | 12 (16.4) | 1 (1.4) |
| 2019/07 | 52 | 33 (63.5) | 16 (30.8) | 5 (9.6) | 12 (23.1) | 4 (7.7) | 2 (3.8) | 1 (1.9) | 7 (13.5) | 19 (36.5) | 5 (9.6) | 15 (28.8) | 2 (3.8) |
| 2019/08 | 58 | 36 (62.1) | 15 (25.9) | 5 (8.6) | 11 (19.0) | 6 (10.3) | 2 (3.4) | 0 (0) | 5 (8.6) | 26 (44.8) | 3 (5.2) | 22 (37.9) | 2 (3.4) |
| 2019/09 | 66 | 44 (66.7) | 26 (39.4) | 2 (3.0) | 24 (36.4) | 9 (13.6) | 3 (4.5) | 4 (6.1) | 12 (18.2) | 23 (34.8) | 5 (7.6) | 20 (30.3) | 2 (3) |
| 2019/10 | 63 | 38 (60.3) | 22 (34.9) | 4 (6.3) | 18 (28.6) | 3 (4.8) | 0 (0) | 2 (3.2) | 13 (20.6) | 20 (31.7) | 5 (7.9) | 17 (27.0) | 0 (0) |
| 2019/11 | 65 | 39 (60.0) | 21 (32.3) | 2 (3.1) | 19 (29.2) | 10 (15.4) | 2 (3.1) | 4 (6.2) | 4 (6.2) | 26 (40.0) | 8 (12.3) | 24 (36.9) | 0 (0) |
| 2019/12 | 50 | 26 (52.0) | 16 (32.0) | 4 (8.0) | 12 (24.0) | 4 (8.0) | 3 (6.0) | 5 (10) | 1 (2.0) | 15 (30.0) | 1 (2.0) | 14 (28.0) | 0 (0) |
| 2020/01 | 65 | 41 (63.1) | 23 (35.4) | 8 (12.3) | 16 (24.6) | 6 (9.2) | 3 (4.6) | 5 (7.7) | 3 (4.6) | 19 (29.2) | 5 (7.7) | 15 (23.1) | 0 (0) |
| 2020/02 | 65 | 29 (44.6) | 15 (23.1) | 4 (6.2) | 11 (16.9) | 6 (9.2) | 0 (0) | 0 (0) | 5 (7.7) | 19 (29.2) | 2 (3.1) | 17 (26.2) | 2 (3.1) |
| 2020/03 | 65 | 28 (43.1) | 12 (18.5) | 0 (0) | 12 (18.5) | 5 (7.7) | 4 (6.2) | 1 (1.5) | 2 (3.1) | 18 (27.7) | 6 (9.2) | 14 (21.5) | 1 (1.5) |
| 2020/04 | 63 | 21 (33.3) | 3 (4.8) | 0 (0) | 3 (4.8) | 4 (6.3) | 0 (0) | 1 (1.6) | 0 (0) | 20 (31.7) | 2 (3.2) | 19 (30.2) | 1 (1.6) |
| 2020/05 | 34 | 16 (47.1) | 2 (5.9) | 0 (0) | 2 (5.9) | 2 (5.9) | 0 (0) | 0 (0) | 0 (0) | 16 (47.1) | 3 (8.8) | 15 (44.1) | 1 (2.9) |

(): positivity rate, defined as percentage of confirmed cases of all examined patients. NIE: non-influenza enveloped virus; PIV: parainfluenza; hMPV: human metapneumovirus; sCoV: seasonal coronavirus; RSV: respiratory syncytial virus. AdV: adenovirus; EnV/RhV: enterovirus/rhinovirus; hBoV: human bocavirus.
*: calculated as patients being infected with at least one type of NCRVs.
**: calculated as patients being infected with at least one of the four viruses (PIV, hPMV, sCoV, and RSV).
